# Pattern of treatment of behavioural and psychological symptoms of dementia and pain: evidence on pharmacoutilization from a large real-world sample and from a centre for cognitive disturbances and dementia

**DOI:** 10.1007/s00228-020-02995-w

**Published:** 2020-09-15

**Authors:** Damiana Scuteri, Marilù Vulnera, Brunella Piro, Roberto Bruno Bossio, Luigi Antonio Morrone, Giorgio Sandrini, Stefano Tamburin, Paolo Tonin, Giacinto Bagetta, Maria Tiziana Corasaniti

**Affiliations:** 1 Pharmacotechnology Documentation and Transfer Unit, Section of Preclinical and Translational Pharmacology, Department of Pharmacy, Health and Nutritional Sciences, University of Calabria, Rende, Italy; 2 Pharmacovigilance Territorial Service, Pharmaceutical Department, Provincial Health Authority, Cosenza, Italy; 3 Center for Diagnosis and Cure of Dementia, Neurology Service of the Health District of Cosenza, Provincial Health Authority, Cosenza, Italy; 4 Department of Brain and Behavioural Sciences, IRCCS C. Mondino Foundation Neurologic Institute, University of Pavia, Pavia, Italy; 5 Department of Neurosciences, Biomedicine and Movement Sciences, University of Verona, Verona, Italy; 6 Regional Center for Serious Brain Injuries, S. Anna Institute, Crotone, Italy; 7 Department of Health Science, University Magna Graecia, 88100 Catanzaro, Italy

**Keywords:** Alzheimer’s disease, BPSD, Pain, Antipsychotics, Antidepressants, Analgesics

## Abstract

**Purpose:**

Data concerning the number of diagnoses and of the drugs prescribed to patients affected by dementia are still scarce. Here we test whether or not (1) prescription of symptomatic drugs against Alzheimer’s disease (AD) may approximate the number of patients affected by dementia in Italy and (2) adherence to this treatment affects the pattern of prescription of drugs (i.e. antipsychotics and antidepressants) for behavioural and psychological symptoms of dementia (BPSD) and the previously reported limited prescription of analgesics.

**Methods:**

This retrospective observational study concerns 84,235 subjects older than 60 years and registered in the provincial prescription database of the health district of Cosenza accounting for a population of 298,000 inhabitants. The prescribing pattern of antipsychotics, antidepressants, and analgesics has been investigated in patients receiving concurrent prescriptions of acetylcholinesterase inhibitors (AChEI) and/or memantine. Data from a single centre for cognitive disturbances and dementia (CDCD) in the same health district were used to explore at which stage dementia was diagnosed. The study was approved by Calabria Region Ethical Committee no. 31/2017 and registered on October 31, 2017.

**Results:**

The data show that 859 patients are treated with AChEI and/or memantine; 420 patients (48.89%) receive at least 80% of the recommended medications. CDCD data indicate a delay in dementia diagnosis, which often was made when the patients were moderately to severely demented (Mini Mental State Examination, MMSE ≤ 20). Adherence did not influence prescription of most of the drugs explored, but use of non-steroidal anti-inflammatory drugs was higher in non-adherent patients. Antipsychotics and antidepressants are frequently used (20.61–20.71% and 42.37–51.43%, respectively), and this, at least in part, might stem from the observed under-treatment of chronic pain (opioids are prescribed in the 4.76% and 12.46% of adherent and non-adherent patients and gabapentin and pregabalin are used in the 4.29% and 4.07% of adherent and non-adherent patients respectively), resulting in more frequent BPSD. 16.43% of patients receive antipsychotics for longer than 6–12 weeks.

**Conclusion:**

This 2-year period study, including a wide cohort of community demented patients, shows that dementia is diagnosed late and that prevalence of BPSD prescriptions is high and not impacted by adherence to anti-dementia drugs. The rate of prescription of potentially harmful antipsychotics and antidepressants appears to be high though whether the concomitantly observed limited prescription of analgesics might be a contributing factor needs to be further investigated. Our data support the development of strategies to improve the management of BPSD.

## Introduction

Fifty million people suffer from dementia worldwide, and the most common form is represented by Alzheimer’s disease (AD) [1]. The lack of a definite etiopathogenesis makes the discovery of disease-modifying drugs difficult (see [2, 3]). Several factors contribute to delayed diagnosis of dementia [4, 5], and this means the two-thirds of all cases go missed in primary care [6, 7]. Moreover, the complexity and individual variability of the clinical manifestations of dementia and the paucity of treatment options, among other factors, prompt the occurrence of appropriate symptomatic strategies [6, 8].

The large majority (97%) of patients suffering from dementia develops neuropsychiatric symptoms [9–11], consisting in disturbances of behaviour, mood, thought, and perception (see [12]), known as behavioural and psychological symptoms of dementia (BPSD). Treatment of BPSD is a hard challenge, and it is based on the approved use of the atypical antipsychotic risperidone for no longer than 6–12 weeks (see [13]). The use of antipsychotics in demented patients is accompanied by an increased risk of mortality [14]. Quite importantly, chronic pain afflicts 72% of the oldest old (i.e. over 85 years old) patients [15, 16]. In particular, 60–80% of demented patients living in nursing homes are estimated to suffer from pain [17]. The impaired communication skills of patients affected by severe dementia limit their self-report of pain, thus causing under-detection and under-treatment of pain, often contributing to BPSD and agitation [18, 19]. Moreover, it is even more difficult to separate different types of pain, e.g. neuropathic vs nociceptive [17]. Accurate analgesic treatment provides reduction of agitation, and the treatment of pain can reduce the use of unnecessary neuroleptics in demented patients [19]. BPSD include depressive symptoms that can be linked to unrelieved pain. Incidentally, persistent and chronic pain per se represents risk factors for cognitive decline and dementia (see [20–22]). Previous work from our group has demonstrated accuracy of treatment of inflammatory pain, but limited access to the treatment of chronic and neuropathic pain in demented patients in Calabria in the period 2014–2016 [23, 24].

Here we report the results of a retrospective observational study (2-year period ranging from 2017 to 2018). The use of antipsychotics, antidepressants, and analgesics was investigated in patients receiving concurrent prescriptions of acetylcholinesterase inhibitors (AChEI) and/or memantine; the data were derived from a database of a provincial health district accounting for a population of 298,000 inhabitants. Under these experimental conditions, here we also report the pattern of prescription of analgesics because, despite the above described relationship between pain and BPSD, little data exist in the literature. In addition, data from a single centre for cognitive disturbances and dementia (CDCD) in the same health district were used to explore at which stage dementia was diagnosed.

## Materials and methods

### Design of the study

This retrospective observational study has been carried out in collaboration with the Calabrian pharmacovigilance territorial service and the CDCD of the provincial health district of Cosenza (Calabria, Italy). Anonymized data have been extracted from the regional drug reimbursement and prescription database including all the prescriptions of drugs reimbursed by the National Health System (NHS). Collected data are a comprehensive reflection of dementia patients on the basis of anti-dementia symptomatic treatment. Over-the-counter purchased drugs and drugs prescribed in dosages or indications not reimbursed by the NHS are not included in the analysis. Information about AD severity and other comorbidities, education, and social status are not available in regional drug reimbursement and prescription database. In particular, available information for each patient include age, date of birth, sex, province and prescribing district, date and number of prescriptions for Anatomical Therapeutic Chemical (ATC) code, dispensation, number of boxes, active principles, and medical specialty. The health district includes a population of 298,000 inhabitants, of whom 84,235 people older than 60 years represent those most affected by dementia. The need for written informed consent was waived owing to the use of anonymized data only. The study was conducted in accordance with the Declaration of Helsinki, and the protocol was approved (no. 31/2017) by the Ethics Committee, Section for Northern Calabria, Calabria Region, 87100, Cosenza, Italy.

This study has examined the prescriptions of AChEI (donepezil, rivastigmine, and galantamine) and memantine (N06D, according to the ATC), provided by the Regional Health Service through territorial pharmacy and by direct distribution for the 2-year period 2017–2018. This treatment identifies unequivocally patients suffering from AD, on the basis of the note 85 set by the Italian Drug Regulatory Agency (AIFA). The latter note states that prescriptions of AChEI and memantine are registered in the database for reimbursement only for patients affected by AD; in particular, the prescriptions are limited to (1) donepezil, rivastigmine, and galantamine for mild AD with MMSE between 21 and 26; donepezil, rivastigmine, galantamine, and memantine for moderate AD with MMSE between 10 and 20. Switch among AChEIs and memantine was not further specified in the database. In particular, two a priori hypotheses have been formulated and tested:

1. we have hypothesized that the number of patients receiving prescriptions of symptomatic drugs against AD in this real-world setting may approximate the number of patients affected by dementia in Italy [25].
2. we have hypothesized that adherence to the symptomatic treatment of AD affects the pattern of prescription of drugs for BPSD and the previously reported [23, 24] limited prescription of analgesics.

To test these hypotheses, the prescriptions of antipsychotics (chlorpromazine, levomepromazine, fluphenazine, periciazine, haloperidol, pimozide, clozapine, olanzapine, quetiapine, clotiapine, amisulpride, levosulpiride, risperidone, and aripiprazole), antidepressants (clomipramine, trimipramine, amitriptyline, fluoxetine, citalopram, paroxetine, sertraline, fluvoxamine, escitalopram, trazodone, mirtazapine, venlafaxine, reboxetine, duloxetine, and vortioxetine), and analgesics have been monitored and analyzed for each individual patient receiving pharmacological treatment for AD. Moreover, MMSE of patients referred to CDCD of the provincial health district of Cosenza has been studied to gain insight on the occurrence of delay for referral to the CDCD of patients with suspected dementia. This subgroup of the population has been stratified for sex and age, and the percentage of treatment with psychotropic drugs has been analyzed.

### Data analysis

Data have been extracted from the database and analyzed through Microsoft Office Excel 2010 (Microsoft, Milan, Italy). Statistical analyses have been performed using GraphPad Prism® 6.0 (GraphPad software Incorporated, San Diego, CA, USA). The results have been evaluated statistically for difference using *χ*^2^ test for categorical variables considering *p* < 0.05 significant.

## Results

### Stratification of the patients

The database searched for the study belongs to a health district including 298,000 inhabitants, of whom 84,235 over 60 years of age. Within the analyzed population of 84,235 individuals, 859 (1.02%) patients older than 60 years (females = 513 [59.70%]; males = 346 [40.30%]) receive treatment with AChEI and memantine.

The symptomatic dementia treatment is as follows: 408 patients (47.50%) are treated with AChEI, 298 patients (34.70%) with memantine, and 153 (17.80%) receive both AChEI and memantine. Data on the prescriptions of AChEI and memantine in the sample studied within the 2-year period 2017–2018 show that 420 patients (0.49% of the total sample of 84,235 individuals and 48.89% of the whole population receiving dementia treatment) are adherent to the treatment. Among these, 190 patients (45.24% of the adherent patients) are treated with AChEI, 112 (26.66%) with memantine, and 118 (28.10%) with both AChEI and memantine. The stratification of patients is reported in Table 1.

**Table 1:** Stratification of the patients

	Patients	AChEI	Memantine	Both AChEI and memantine
Total sample	298,000			
Over 60	84,235			
Treated for dementia	859 (346 males and 513 females)	408	298	153
Adherent to treatment	420	190	112	118

Eight hundred fifty-nine patients (1.02% of the over 60 population considered; 346 males and 513 females) are treated for dementia in a sample of 298,000 inhabitants (84,235 over 60). In particular, 408 patients (47.50%) are treated with acetylcholinesterase inhibitors (AChEI), 298 patients (34.70%) with memantine, and 153 (17.80%) receive both AChEI and memantine. Of these, 420 are adherent to the treatment: 190 patients (45.24% of the adherent patients) are treated with AChEI, 112 (26.66%) with memantine, and 118 (28.10%) with both AChEI and memantine.

The subgroup of this population (103 patients) referred to the CDCD of the health district of Cosenza has been studied confirming the prevalence of females among demented patients (Table 2); 68.93% of the whole sample is females. The majority of patients (62.14%) comes to clinic observation when the Mini Mental State Examination (MMSE) score is ≤ 20 (Table 2). In this population, 19.41% and 8.73% of patients are prescribed antipsychotics and antidepressants, respectively.

**Table 2:** Clinical features and treatments of 103 patients referred to the CDCD of Cosenza

	Males	Females	MMSE ≤ 20	Receiving treatment with antipsychotics	Receiving treatment with antidepressants
% of patients	31.07	68.93	62.14	19.41	8.73

Dementia is more common in females (68.93%). Among them, 62.14% of patients comes to clinic observation at a stage of moderate-to-severe dementia (MMSE ≤ 20). 19.41% of patients receives prescriptions of antipsychotics, and 8.73% of them is prescribed antidepressants.

### Treatment patterns of patients who are adherent/non-adherent to the therapy with AChEI and memantine

**Fig. 1:**
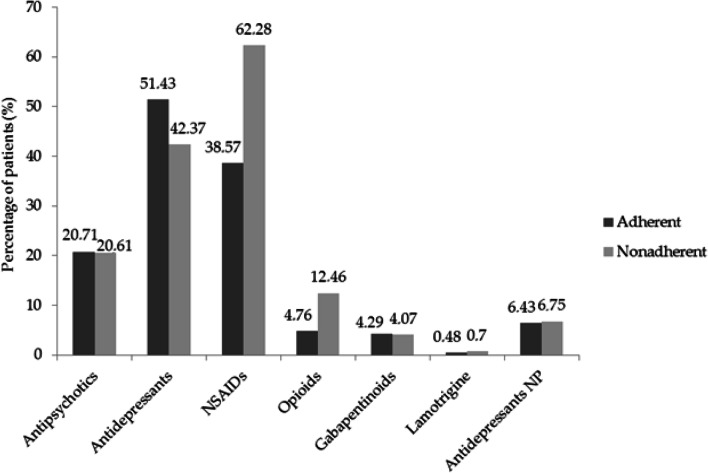
Difference in treatments between patients adherent and non-adherent to the therapy with AChEI and memantine. The use of antipsychotics is almost superimposable in the adherent (20.71%) and non-adherent (20.61%) patients. Antidepressants are extensively used (non-adherent patients: 42.37%; adherent patients: 51.43%). The treatment with NSAIDs presents values of 38.57% for adherent and of 62.28% for non-adherent patients. Opioids are used in 4.76% and 12.46% of adherent and non-adherent patients, respectively. Gabapentinoids are used in the 4.29% and 4.07% of adherent and non-adherent patients, respectively. 0.48% of adherent and 0.7% of non-adherent patients are treated with lamotrigine. 6.43% of adherent and 6.75% of non-adherent patients receive treatment with antidepressants used for neuropathic pain (NP). Data are expressed as percentages and evaluated statistically for difference considering *p* < 0.05 significant (*χ*^2^ test: *p* = 0.1204)

We compared the frequency of use of drugs for BPSD (i.e. antipsychotics and antidepressants) and for pain (i.e. non-steroidal anti-inflammatory drugs (NSAIDs), opioids, gabapentinoids, lamotrigine, amitriptyline and duloxetine) in patients who were adherent vs those non-adherent to the therapy with AChEI and memantine (Fig. 1).

The results demonstrate that the pattern of antipsychotics use is almost superimposable in the two populations, i.e. adherent (20.71%) and non-adherent (20.61%) patients (Fig. 1). In particular, data concerning the prescriptions of antipsychotics in the adherent cluster have been analyzed for each patient, unraveling that 16.43% of these patients are treated for longer than 6–12 weeks. Antidepressants are extensively used in non-adherent patients (42.37%) and even more in the adherent ones (51.43%) (Fig. 1). Among antidepressants, the prescriptions of amitriptyline and duloxetine have been considered separately, since they could be used to treat both depression and/or neuropathic pain but are more commonly prescribed for the latter condition in Italy. These two drugs are used in 6.43% of adherent and 6.75% of non-adherent patients (Fig. 1).

The treatment with NSAIDs differs more than the others between adherent and non-adherent patients, showing values of 38.57% and 62.28%, respectively (Fig. 1). Opioids are prescribed in 4.76% and 12.46% of adherent and non-adherent patients, respectively. There is a small amount of patients receiving treatment for neuropathic pain: gabapentin and pregabalin are used in 4.29% and 4.07% of adherent and non-adherent patients, respectively; lamotrigine is prescribed to 0.48% of adherent and 0.7% of non-adherent patients; 6.43% of adherent and 6.75% of non-adherent patients are treated with amitriptyline and duloxetine (Fig. 1). Difference between adherent and non-adherent patients for the whole dataset does not reach statistical significance (*p* = 0.1204).

## Discussion

Dementia has a remarkable social burden on the global population, and data from real-world settings of community are still very scarce. Our results highlight an under-treatment of dementia since only 859 individuals receive treatment with AChEI and memantine, in comparison with the number of patients affected by dementia in Italy, i.e. 900 to < 1000 per 100,000 inhabitants [25], accounting for 2700–3000 potential cases in the population we studied. These figures, however, are in keeping with reports from other European countries [26, 27]. Indeed, dementia is often underdiagnosed by clinicians or underreported by family, determining a rate of about 50% of people with dementia really receiving a diagnosis [28]. In particular, the rate of undetected dementia reaches up to 61.7% with variability among Europe, the USA, and India [29]. The underdiagnosis of dementia is often due to the severity of cognitive impairment, advanced age, and low educational level especially in rural areas [29]. The view that the limited number of patients diagnosed with dementia is one of the reasons of under-treatment is in accordance with our data from the CDCD of the same health district, where 62.14% of patients were referred with a MMSE ≤ 20 underscoring a clinical condition of moderate-to-severe dementia. These data may stem from the lack of a healthcare diagnostic and therapeutic strategy in Calabria, a condition that, actually, occurs in the majority of Italian Regions (i.e. 13 out of 20 [30]). A longitudinal retrospective cohort study conducted in the UK from 2005 to 2015 [31] shows an increase in diagnosis and treatment of dementia and a decrease in the prescription of antipsychotics following the introduction of the UK National Dementia Strategy [31]. A recent study analyzed the prescribing rates since the launch of the symptomatic drugs for the dementia in 1997 up until 2016, reporting the factors influencing the treatment with AChEI and memantine; the latter are considered linked to regulatory strategies but not always in a predictable way [32]. This strengthens the need for national policies to improve the care of patients suffering from dementia. Another reason for the difference between the estimated number of demented people and those prescribed with AChEI and memantine is that these drugs are reimbursed by the NHS only for patients with MMSE > 10.

An important issue is the non-adherence to the therapy with AChEI and memantine, a serious problem since it is responsible for inadequate treatment of cognitive decline and exacerbation of BPSD [13]. According to WHO, the definition of adherence is the extent to which a person’s behaviour, taking medication, corresponds with agreed recommendations from a health care provider [33]. Adherence to treatment is defined as percentage of prescribed medication taken, and according to the most frequently used cutoff of 80%, the degree of a medication use corresponding or aligning with the recommendations of the prescriber and missing 20% or more of the medication defines non-adherence [34–38]. Conceivably, adherent patients are subjected to more frequent physical examination which can include pain assessment. Indeed, a link between accurate medical care and medication adherence has been found [39]. Our data demonstrate the lack of accurate adherence to dementia treatment, in that only 420 patients out of 859 patients receiving anti-dementia treatment (48.89%) have been found adherent. Non-adherence and discontinuation of the medication in dementia increases the risk of hospitalization and death [40]. In line with a previous European study reporting disease progression on cognitive or functional scales to be among the factors reducing adherence to anti-dementia drugs [41], we may speculate that non-adherence might be related to delayed dementia diagnosis in our sample. However, AChEI and memantine are often prescribed, sometimes inappropriately, in patients with vascular dementia, synucleinopathies, frontotemporal dementia, and other conditions, this point representing a potential bias. Prescription of antipsychotics in our patients, either adherent or non-adherent, is in the lower part of the range (i.e. 20–30%) reported in previous studies [8], likely because we recruited patients in the community rather than in long-term care settings [8]. However, the rate of use of antidepressants is noteworthy (42.37% of non-adherent patients and 51.43% of adherent patients). Difference between adherent and non-adherent patients in this context does not reach statistical significance, suggesting that adherence to symptomatic anti-dementia drugs does not impact the prescriptions of the latter drugs. Memantine is associated to some efficacy in agitation, which however is controversial [42]; in particular, there is an effect on the development of new agitation, but no evidence on already existing agitation [43]. Indeed, the use of anti-dementia drugs, in particular memantine, has been associated with fewer BPSD [44, 45] and a reduced rate of slope for monthly use of antipsychotics [46, 47]. The latter evidence is in line with clinical trials [48, 49] and Cochrane meta-analysis [43] showing the effect of this drug on the development of agitation. However, the stronger effect of memantine as compared with AChEI is seen in an advanced stage of dementia where BPSD are often more severe [46]. At variance with the latter, evidence from clinical trials does not support the efficacy of AChEI and memantine in the treatment of depressive symptoms associated to dementia [50]. Unfortunately, antipsychotics have been often used for more than 6–12 weeks in our sample, increasing their potential for toxicity [13]. A percentage of antidepressants in this population might have been prescribed for neuropathic pain. A high rate of use of antidepressants has been described also in other European countries [51–55]. This aspect has a fundamental importance because, despite the potential of antidepressants for neurogenesis [56, 57], the use of drugs endowed with anticholinergic burden can worsen cognitive decline, increase the risk of falls, institutionalization, and mortality [58–65]. Therefore, antidepressants in demented patients should be prescribed only after appropriate diagnosis and with regular follow-up, since the efficacy and safety of these drugs still awaits support from large, rigorous clinical trials [66, 67]. Psychotropic polypharmacy has been described also in Denmark. Indeed, an overlapping treatment with at least 2 psychotropic drugs has been reported in 25.3% of demented patients in a Danish population-based study [68]. Interestingly, our data demonstrate that patients who are referred to CDCD receive fewer prescriptions; indeed, only 19.41% and 8.73% of patients are prescribed antipsychotics and antidepressants, respectively, and this may stem from a better control of dementia and regular follow-up. Pain could not be recorded in our samples because of the retrospective design of the study and the difficulty of measuring pain in demented patient. However, comparison of our data with previous reports from the UK, where strong opioids are used in 83.9% of non-cancer patients (mean age 67.1 ± 17.0 years) [69], suggests chronic pain to be under-treated in both adherent and non-adherent patients, despite a larger percentage of non-adherent patients being prescribed NSAIDS. Also, the percentage of patients receiving treatment for neuropathic pain is low and, to the best of our knowledge, this is the first study performed on a very large sample including community patients that has highlighted the prescribing attitude towards drugs for neuropathic pain treatment in patients suffering from dementia. This is in line with international epidemiological research showing that demented patients are prescribed fewer pain medications than cognitively intact aged patients [17]. Some patients were under antiepileptic drugs, and epilepsy may occur in demented patients. However, gabapentin and pregabalin are rarely used to treat epilepsy very seldom because of their poor efficacy, while lamotrigine is slow to titrate and not practical in a demented patient.

The above described issues in the management of pain in patients affected by severe dementia are likely due to the lack of an accurate diagnosis because of their reduced communication skills. Moreover, the paucity of studies investigating pharmacodynamics, efficacy, and safety of drugs in demented patients results in poor pain treatment [17]. Therefore, clinical trials evaluating the various treatments used in patients suffering from dementia are needed. Moreover, the adoption of national policies to handle dementia and pain in cognitively impaired patients are mandatory to improve pain assessment and the treatment of BPSD, reducing the off label use of unnecessary psychotropic drugs. Non-pharmacological treatments for the management of BPSD have proven efficacy devoid of the toxicity of atypical antipsychotics [13]. Among others, aromatherapy with Melissa has provided evidence for reduction of agitation [70]. Interestingly, the essential oil of bergamot is endowed with powerful analgesic (see [12, 71, 72]) and anxiolytic, non-benzodiazepine-like properties [73, 74] forming the rational basis for a starting clinical trial (NCT04321889) exploring whether it may represent a safe and effective treatment of BPSD.

Limitations of the present study include the retrospective design and the inclusion of a single health district that may not be representative of the whole Italian population though it might form the rational basis for regional regulatory intervention. Moreover, the regional drug reimbursement and prescription database does not include over-the-counter purchased drugs and drugs prescribed in dosages not reimbursed by the NHS. Future multicenter studies will overcome these limitations.

## Data Availability

All the dataset created are reported in this manuscript.
